# Aging Mechanism of a Diatomite-Modified Asphalt Binder Using Fourier-Transform Infrared (FTIR) Spectroscopy Analysis

**DOI:** 10.3390/ma12060988

**Published:** 2019-03-26

**Authors:** Peng Zhang, Qinglin Guo, Jinglin Tao, Dehua Ma, Yedan Wang

**Affiliations:** 1 JSTI Group Co. Ltd., Nanjing 211112, China; zhpjsits@163.com; 2 National Engineering Laboratory for Advanced Road Materials, Nanjing 211112, China; 3 School of Civil Engineering, Hebei University of Engineering, Handan 056038, China; 4 Jiangxi Transportation Institute, Nanchang 330200, China; taojljxits@163.com; 5 School of Environmental and Biological Engineering, Nanjing University of Science and Technology, Nanjing 210094, China; 6 College of Transportation, Jilin University, Changchun 130025, China; wangydjlu@163.com

**Keywords:** fourier-transform infrared spectroscopy, aging resistance, diatomite-modified asphalt, functional groups

## Abstract

In this paper, Fourier-transform infrared (FTIR) spectroscopy was used to evaluate the effects of diatomite on aging properties of an asphalt binder. The modified asphalts included 5%, 10%, and 15% diatomite, and were prepared in the laboratory. The changes in functional groups of asphalt were employed to investigate the aging mechanisms of the modified and control asphalts. Effects of diatomite on the anti-aging properties of asphalt were analyzed via the changes in intensity of the absorption peaks. Results showed that there were no new functional groups generated after diatomite mixing with asphalt. This indicated that the process of diatomite modification was just physical mixing. Furthermore, parts of saturates and aromatics were volatilized in the aging process of modified asphalt. Polar molecules reacted with oxygen in aging. Meanwhile, carbonyl (C=O) and sulfoxide (S=O) were also generated. The aging resistance of modified asphalt was the best when the diatomite content was 10%. The work of this paper may provide a new perspective to evaluate asphalt aging.

## 1. Introduction

Asphalt concrete is the favorable pavement material in engineering. In the mixing and compacting processes of asphalt mixture, a thermal oxidative reaction, called short-term aging, occurs in the asphalt binder as a result of oxidative aging [1]. In its service, asphalt will harden and become brittle because of the environmental factors and traffic load, which are long-term aging processes. The mechanical properties of the asphalt mixture will be weakened by the effect of aging, until it cannot sustain the traffic load. As a consequence, aging has become a vital factor influencing the durability of asphalt pavement. In order to improve the service life of asphalt pavement, it is necessary to investigate the aging mechanisms of neat and modified asphalt. Furthermore, investigation is instructive for the design of asphalt pavement in the future.

The asphalt macro-properties are determined by its microstructure. Weight loss (volatilization) and oxidation are two main causes of asphalt aging. During the aging process, the naphthene aromatics and saturates are partly converted to asphaltenes, which result in the formation of oxygen-containing functional groups [2,3,4]. Therefore, analysis of the changes of composition, structure, and morphology in asphalt aging by chemical and thermodynamic methods can essentially reveal the aging mechanism of asphalt. Also, the anti-aging mechanism of modifiers can be determined. With the development of chemical analysis technology, there are numerous methods that can be used. Researchers can analyze the changes of asphalt, before and after aging, in the molecular crystal structure by X-ray diffraction (XRD) [5]. The micro morphology of neat and modified asphalt can be observed through scanning electron microscopy (SEM), atomic force microscopy (AFM), and fluorescence microscopy (FM) [6]. The aging degree and rate of asphalt could be obtained using Fourier-transform infrared (FTIR) spectroscopy via analyzing the reaction of functional groups in the molecule [7]. The molecular isomerization, internal cross-linking, and dehydrogenation reactions in the asphalt aging process can be demonstrated by the chemical structure and molecular weight change, observed with gel permeation chromatography (HP-GPC) and nuclear magnetic resonance (NMR) spectrum, respectively [8]. The technique that is most widely used is FTIR because of its sensitivity and operability. It obtains information regarding aliphaticity, aromaticity, and oxygenation rate quickly and reliably [9,10]. This technique can provide more accurate data such as the average distribution length of aliphatic chains, oxygenation, and substitution mode of aromatics [11,12].

Diatomite is a kind of natural inorganic material with a low cost and huge abundance, which has been used in industry as filler, filtering agent, adsorbent, clarifier, and insulator. Diatomite is a sedimentary rock composed of the fossilized skeletons of diatoms, one-celled algae-like plants that accumulate in marine or lacustrine environments [13]. Kietzman et al. [14] first studied the paving performance of diatomite-modified asphalt concrete and pointed out that the high temperature deformation resistance of diatomite-modified asphalt concrete was improved. Cong et al. [15,16] investigated the physical properties, chemical properties, and dynamic rheological properties of diatomite-modified asphalt. Results showed that the viscosity of asphalt increased with increasing diatomite, while the improvement was marginal at low temperature. Diatomite-modified asphalt had better long-term aging resistance. The low temperature performances of diatomite-modified asphalt and asphalt mixture were given by Tan et al. [17]. Their results showed that diatomite-modified asphalt mixtures performed better than the neat mixture at low temperature. Song et al. [18] studied the action process between diatomite and asphalt compositions as well as the enhancement mechanism of travelling performance. Results suggested that diatomite with a porous structure could effectively adsorb the lower molecular group and lower polar aromatic molecules, resulting in the formation of anchorage points which improved the property of the asphalt binder. Cheng et al. [19] determined physical and mechanical properties of diatomite-modified asphalt binders. They found that diatomite powder can significantly improve the high- and low-temperature performances of asphalt. It not only had excellent cracking resistance, but also could withstand greater distortions in low temperature. Guo et al. [20] studied the pavement performance of diatomite glass fiber-modified asphalt mixture through a wheel-tracking test, a low-temperature indirect tensile test, a fatigue test, and a stiffness modulus test. Results showed that the stiffness modulus of asphalt significantly increased after adding diatomite, and when combined with fiberglass, the low temperature property of asphalt improved effectively. Although research on the paving performance of diatomite-modified asphalt mixtures and modified mechanisms was involved, the reaction mechanism between asphalt and diatomite is still unclear. Therefore, systematic investigation on the modified mechanism of diatomite-modified asphalt is helpful in understanding the enhancement process of diatomite-modified asphalt mixtures. In addition, the application of FTIR spectroscopy will provide a quick and convenient method to determine the aging resistance of neat and modified asphalt. In this way, many experiments may be saved.

In this paper, the mechanism of diatomite-modified asphalt (DMA) and aging resistance were revealed by the analysis of functional groups using FTIR spectroscopy. Meanwhile, the effects of diatomite on the asphalt aging resistance were evaluated quantitatively via the absorption peak-value of diatomite-modified asphalt, and the optimal dosage of diatomite for anti-aging purposes was determined.

## 2. Experimental Methods

### 2.1. Raw Materials

**Figure 1:**
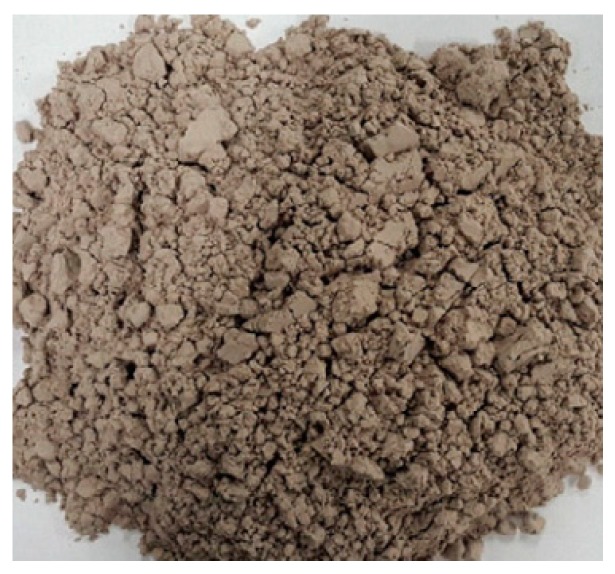
Appearance of diatomite.

The asphalt type AH-90 # from Panjin Petrochemical Industry, Liaoning Province of China was selected in the test. The properties of neat asphalt are given in Table 1. Changbai diatomite is the calcined product obtained from Jilin Province (about 1000 km north of Beijing capital), and its appearance is shown in Figure 1. The corresponding physical properties of diatomite are shown in Table 2 and Table 3.

### 2.2. Methods

#### 2.2.1. Preparation of diatomite-modified asphalt (DMA)

**Figure 2:**
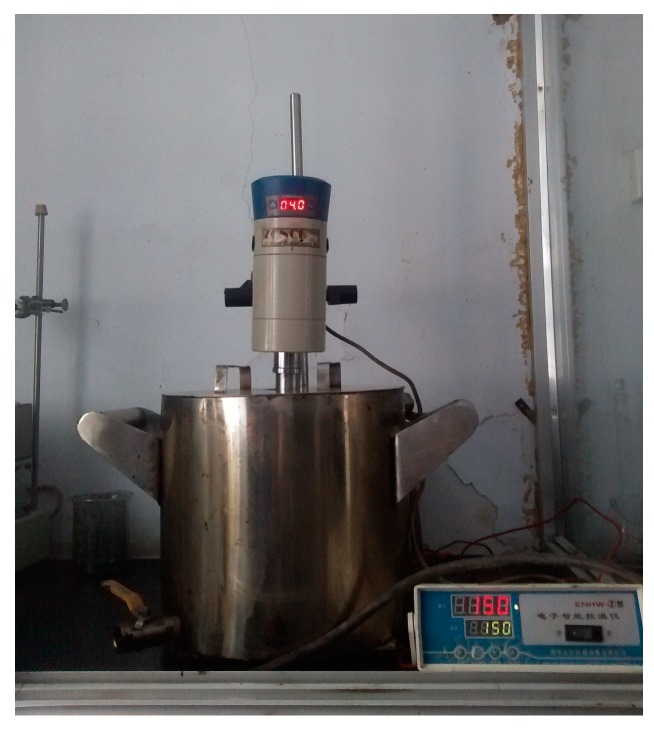
Mixing process of asphalt modification.

The asphalt binder was heated at 150 °C in an oil bath-heating container for 4 h. Then, 5%, 10%, and 15% diatomite (by weight) of asphalt were added, respectively. In order to guarantee an adequate, homogenous mixture, they were mixed for 40 min using high-speed shearing equipment at a speed of 4000 rpm [15,16]. The prepared asphalt binder was stored for future use. The mixing process is shown in Figure 2.

#### 2.2.2. Asphalt Aging Procedure

The thin film oven test (TFOT) was employed to simulate the short-term oxidation that occurs during the mixing process in accordance with ASTM D1754. In this test, asphalt with a weight 50 g was placed in an iron pan, which was set on the tray such that the axis of revolution was vertical. The oven was kept at 163 °C, and the trays were rotated in the oven at a rate of 5.5 rpm for 5 h.

#### 2.2.3. Spectra Sample Preparation

Spectra samples used a potassium bromide crystal smear. A small amount of asphalt (about 5 g) was dissolved in toluene to prepare a 5% solution. The sealed glasses were used to hold the solution. A small drop of solution was taken by a dropper (ensuring the weight of each test specimen was equal) and was put on a potassium bromide chip. When toluene volatilized completely under an infrared lamp, the samples were placed in the FTIR.

## 3. Results and Discussion

### 3.1. Modified Mechanism of DMA Based on Fourier-transform infrared (FTIR) Spectroscopy

**Figure 3:**
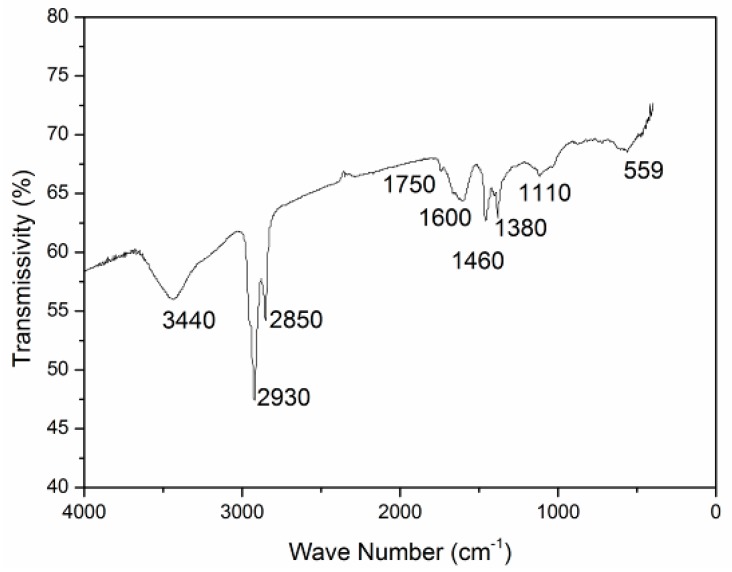
Fourier-transform infrared (FTIR) spectrum of neat asphalt binder.

It is well-known that every organic compound has its own characteristic infrared spectrum, and the chemical molecular structure of asphalt can be obtained through the analysis of functional groups. A spectra image of neat asphalt is shown in Figure 3.

As seen in Figure 3, it can be found that absorption peaks were generated at 3440 cm^−1^, 2930 cm^−1^, 2850 cm^−1^, 1600 cm^−1^, 1460 cm^−1^, and 1380 cm^−1^. The absorption band from 4000 to 1300 cm^−1^ was caused by the stretching vibration, which was called the functional group region. And, except for a single bond stretching vibration, there was also vibration in the region from 1300 to 600 cm^−1^, which was called the fingerprint region. Based on the result of the characteristic absorption frequencies of organic functional groups [20], waves of representative functional groups are obtained. The results are shown in Table 4.

Hence, it can be confirmed from the infrared absorption spectrum that asphalt mainly consisted of alkanes, cycloalkanes, and aromatics and their derivatives.

**Figure 4:**
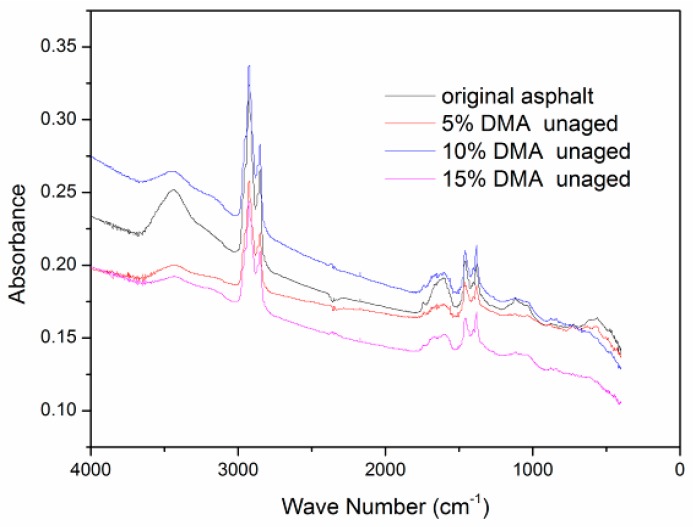
Spectra of neat asphalt and diatomite-modified asphalt (DMA).

Figure 4 shows the FTIR spectra of neat asphalt and binders with 5%, 10%, and 15% of diatomite by weight. It is observed that no new peaks were generated, and the existing absorption peaks had no significant changes. No chemical reaction occurred between asphalt and diatomite; the diatomite merely blended with the asphalt physically. This was consistent with the results obtained by Cong et al. [15]. However, the intensity of the existing absorption peak changed significantly. The peak values of 3440 cm^−1^, 2930 cm^−1^, 2850 cm^−1^, and 1600 cm^−1^ decreased after adding diatomite. The absorption peaks represented the percentages of functional groups in asphalt. In order to evaluate the changes of peaks quantitatively, the absorbance spectra was chosen, and baseline correction was taken for FTIR spectra. The values of the absorption peaks are obtained and summarized in Table 5.

As listed in Table 5, the peak values reduced remarkably with the addition of diatomite. It revealed that the percentages of hydrogen bonds, paraffin, and naphthenic and aromatic compounds decrease. Meanwhile, the molecular hydrogen of diatomite-modified asphalt that included 15% diatomite decreased the largest; it was 20% compared with the neat asphalt. This indicated that the links of asphalt molecules were broken because of high-speed shearing. When the diatomite dosage reached 15%, the content of paraffin was the lowest. And when the dosage was 10%, the largest reduction happened in aromatic compounds. It can be inferred that diatomite adsorbed partial saturates and aromatics in the asphalt, a result of the special porous structure of the diatomite particle.

In addition, it can be concluded that the chemical compositions of asphalt binders changed when they were mixed with diatomite. The peak value of light components decreased with the increasing of diatomite. The alkanes and aromatics declined significantly when the diatomite content was 10%. However, the decline tended to be steady with an increasing content. The reason was that the adsorption action was weakened when the diatomite exceeded the upper limit, owing to the increase in viscosity of the asphalt.

### 3.2. Aging Mechanism of DMA

**Figure 5:**
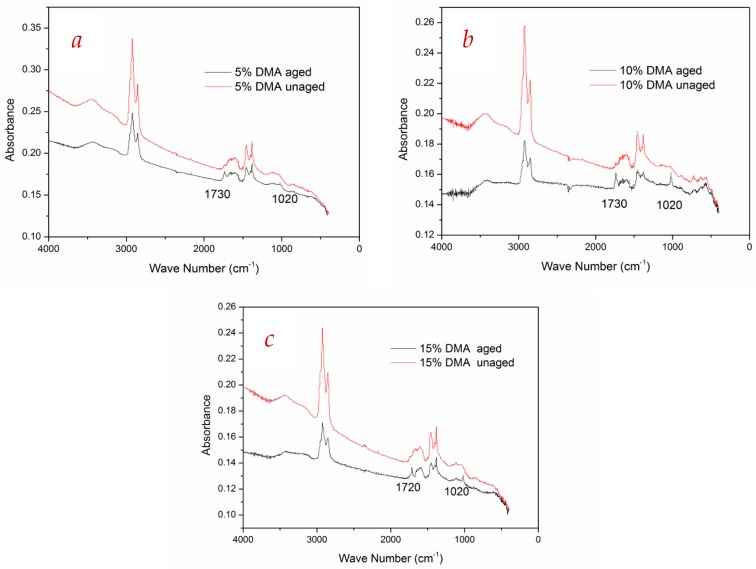
FTIR spectra of DMA. (**a**) 5% DMA; (**b**) 10% DMA; (**c**) 15% DMA.

In order to investigate the aging mechanism of DMA, the peak value difference before and after aging was obtained, which represented the degree of aging. The larger the value of the peak was, the more advanced the aging of DMA was. Spectrums of DMA for three contents (5%, 10%, and 15% by weight) are shown in Figure 5.

As shown in Figure 5, it can be seen that the peak intensities of paraffin, olefins, and aromatics decreased after aging. The detailed differences of peak changes are summarized in Table 6.

**Figure 6:**
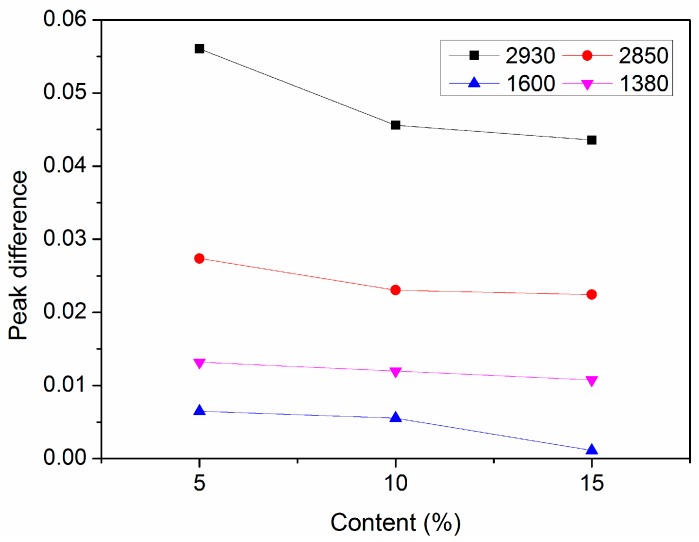
Peak value differences after aging versus diatomite content for functional groups.

Figure 6 shows the trend of the peak difference of functional groups versus diatomite content. As can be seen from Figure 6, the peaks of light components declined after aging, indicating that the light components in the asphalt volatilized during the aging process. The absorption peak intensities of different functional groups changed with the varying dosage of diatomite. The maximum difference was observed for the 5% type, followed by 10%, and 15%. Peak differences for the 10% and 15% types were almost equal before and after aging. Experimental data revealed that diatomite could inhibit volatilizing of light components in the aging process. The porous structure of the diatomite particle is similar to a microcapillary structure, which can produce capillarity. Therefore, it increased the interfacial force between diatomite particles and asphalt, and it prevented volatilization of light components.

Meanwhile, new peaks were generated with wave numbers 1730 cm^−1^ and 1020 cm^−1^ in the spectra of aged asphalt. The peak of 1730 cm^−1^ represented the vibration frequency of the carbonyl of ketones and esters, and the vibration frequency of sulfoxide was 1020 cm^−1^. Herein, the chemical reaction occurred during asphalt aging. A part of unsaturated hydrocarbon groups reacted with oxygen and resulted in the generation of carbonyl and sulfoxide groups. More details of the chemistry supporting the mechanism can be found in the work of Wang et al. [21] and Herrington et al. [22].

In this regard, a part of saturates and aromatics become volatile in the aging process. Active molecules reacted with oxygen in air and generated aging functional groups, including the carbonyl (C=O) and sulfoxide (S=O) groups, in a high-temperature environment.

### 3.3. Diatomite Inhibition of Asphalt Aging

**Figure 7:**
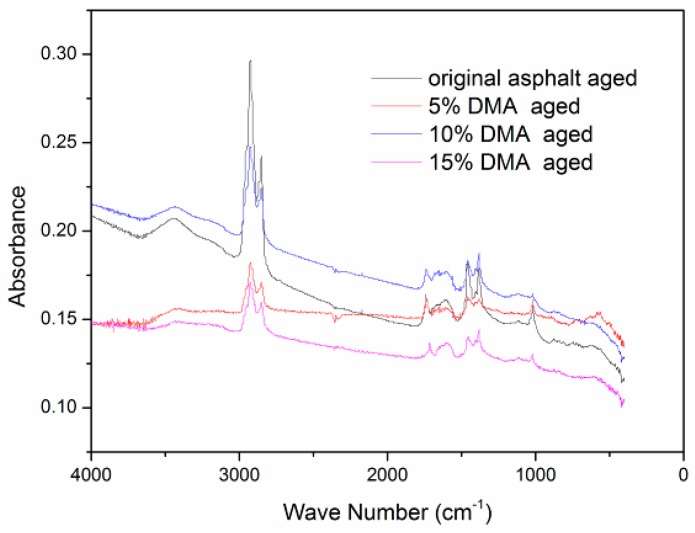
FTIR spectra of aged original asphalt and diatomite-modified asphalt.

For the quantitative evaluation of the anti-aging effects of diatomite in asphalt, the FTIR spectra of neat asphalt and DMA after aging are presented. They are shown in Figure 7.

**Figure 8:**
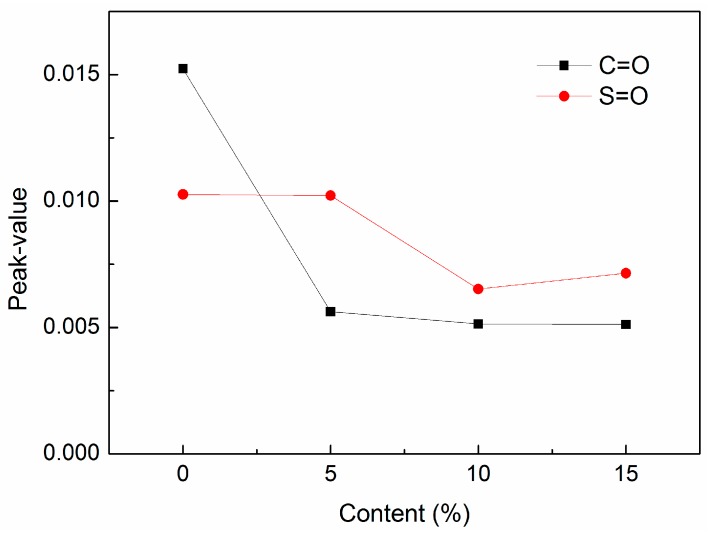
Peak value changes of carbonyl and sulfoxide groups after aging.

In this study, the contents of carbonyl and sulfoxide groups were adopted as indices to analyze the anti-aging action. The changes of carbonyl and sulfoxide groups are described in Figure 8.

It can be found that the contents of carbonyl and sulfoxide of DMA were less than the neat asphalt. And peak values of carbonyl and sulfoxide groups declined with the increase of diatomite. The peak value of the sulfoxide group for the 5% type was almost equal to that of neat asphalt, although the value of the carbonyl group declined. For the type including 10% diatomite, the amount of the carbonyl group was one-third that of neat asphalt, and the amount of the sulfoxide group was nearly one-half that of the neat asphalt. The results proved that asphalt aging can be inhibited by diatomite because of its special porous structure, which can adsorb light components in asphalt. So, it possessed good anti-aging properties. Asphalt aging inhibition increased with diatomite increase. And the trend kept stable when the addition of diatomite went up to 10%.

**Figure 9:**
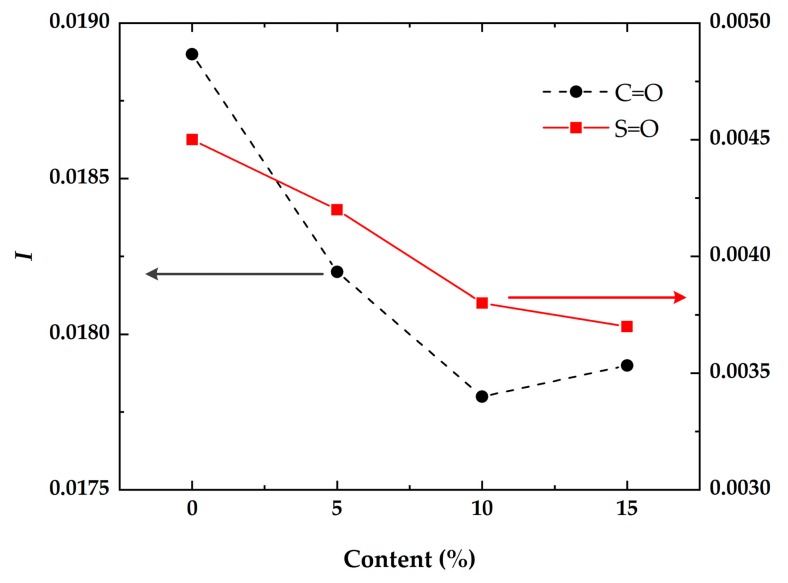
Aging index changes of carbonyl and sulfoxide groups.

According to the method proposed by Mouillet [4] and Petersen et al. [23], diatomite inhibition of asphalt aging was evaluated with *I_C=O_* and *I_S=O_*, which were calculated using the following equations. Results of *I_C=O_* and *I_S=O_* are shown in Figure 9.

(1) $$I_{C=O}=\frac{A_{1730}}{\sum A_{i}},$$

(2) $$I_{S=O}=\frac{A_{1020}}{\sum A_{i}},$$

(3) $$\begin{array}{l} \sum A_{i}=A_{2930}+A_{2850}+A_{1730}+A_{1600}+A_{1460}\\ \;\;\;\;+A_{1380}+A_{1020}+A_{968}+A_{860}+A_{810}+A_{744}+A_{722} \end{array}$$

As seen in Figure 9, it was found that the aging indexes, *I_C=O_* and *I_S=O_*, both decreased with the increase of diatomite. This indicated that the diatomite played an important role in inhibiting asphalt aging. And, the changes of *I_C=O_* and *I_S=O_* gradually flattened out when the diatomite content was more than 10%. This agreed with the changing peak values of the carbonyl and sulfoxide groups.

## 4. Conclusions

Diatomite-modified asphalt (DMA) is prepared using high-speed shearing method. The short-term thermo-oxidative aging mechanism of neat asphalt and DMA were studied using the FTIR technique. The following conclusions can be drawn:

1. Asphalt mainly consisted of alkanes, cycloalkanes, and aromatics and their derivatives. No chemical reaction occurred between asphalt and diatomite when asphalt was blended with diatomite; it was only simple physical mixing. However, alkane and aromatic compounds declined, as the diatomite mixed with asphalt for its absorption action.
2. Alkanes, aromatics, and other lightweight components reduced as new peaks occurred in the FTIR spectra. Chemical reactions occurred during the aging process, and oxygen-containing functional groups were generated.
3. Diatomite can inhibit the aging process of asphalt as a result of its physical adsorption function. Diatomite inhibition of asphalt aging increased quickly when diatomite content was less than 10%. The optimum content of diatomite was 10% by weight of asphalt based on the result of FTIR
4. In summary, diatomite can improve the thermal oxidative aging resistance of asphalt to a certain extent. It is meaningful to construct diatomite-modified asphalt pavement that can prevent the reduction in service life caused by aging.

## Figures and Tables

**Table 1:** Physical properties of neat asphalt.

Property	Test Value	Method
Penetration (100 g, 5 s, 0.1 mm, at 25 °C)	90	ASTM D 5
Softening point *T_R&B_* (°C)	42.6	ASTM D 36
Ductility (15 °C, 5 cm/min, cm)	>100	ASTM D 113
Density (15 °C, g/cm^3^)	1.014	ASTM D 70
Mass loss (%)	0.37	–

**Table 2:** Basic properties of diatomite.

Indexes	Color	Specific Weight (g/cm^3^)	Bulk Density (g/cm^3^)	PH Value
Value	Brown	2.1~2.3	0.35~0.42	7~8

**Table 3:** Particle size distribution of diatomite.

Particle Size (μm)	>40	40~20	20~10	10~5	<5
Percentage (%)	1.4	2.1	4.4	27	62

**Table 4:** Featured functional groups of neat asphalt.

Wave Number (cm^−1^)	Functional Groups
3440	Intermolecular hydrogen bond (O–H) vibration
2930	The antisymmetric stretching vibration absorption band of the alkyl (C–H)
2850	The symmetric stretching vibration absorption band of the alkyl (C–H)
1600	Conjugated double bonds (C=C) stretching vibration in aromatics
1460	The C–H asymmetric deformations in CH2 and CH3 vibrations
1380	The C–H symmetric deformation in CH3 vibrations
1110	The C–O stretching vibration in saturated alcohols
559	The C–H out-plane bending vibrations in unsaturated hydrocarbons

**Table 5:** Peak values of asphalt spectra.

Wave Number (cm^−1^)		3440	2930	2850	1600
Peak values	Neat asphalt	0.031106	0.120366	0.065797	0.018052
	5% DMA	0.012263	0.108763	0.058999	0.012298
	10% DMA	0.008514	0.073873	0.040033	0.006776
	15% DMA	0.006353	0.072573	0.040859	0.008403

**Table 6:** Peak values for the featured functional groups of DMA.

Wave Number (cm^−1^)		2930	2850	1600	1380
5% DMA	Before aging	0.108763	0.058999	0.012298	0.033338
	After aging	0.052721	0.031635	0.005823	0.020169
	Difference	0.056042	0.027364	0.006475	0.013169
10% DMA	Before aging	0.073873	0.040033	0.006776	0.018216
	After aging	0.028276	0.016989	0.001244	0.006258
	Difference	0.045597	0.023044	0.005532	0.011958
15% DMA	Before aging	0.072573	0.040859	0.008403	0.026131
	After aging	0.029026	0.018423	0.007289	0.015366
	Difference	0.043547	0.022436	0.001114	0.010765
